# Contributions of COVID-19 Pandemic-Related Stressors to Racial and Ethnic Disparities in Mental Health During Pregnancy

**DOI:** 10.3389/fpsyt.2022.837659

**Published:** 2022-03-14

**Authors:** Lyndsay A. Avalos, Nerissa Nance, Yeyi Zhu, Lisa A. Croen, Kelly C. Young-Wolff, Ousseny Zerbo, Monique M. Hedderson, Assiamira Ferrara, Jennifer L. Ames, Sylvia E. Badon

**Affiliations:** Division of Research, Kaiser Permanente Northern California, Oakland, CA, United States

**Keywords:** COVID-19, prenatal depression, prenatal anxiety, antenatal depression, antenatal anxiety, stressors, social determinants of health

## Abstract

**Background:**

This study aimed to identify racial and ethnic disparities in prenatal mental health and identify COVID-19 pandemic-related health/healthcare and economic contributors to these disparities, using an established framework for disparity investigation.

**Methods:**

This cross-sectional study includes 10,930 pregnant people at Kaiser Permanente Northern California who completed an online survey between June 22, 2020 and April 28, 2021 on COVID-19 pandemic-related health/healthcare and economic stressors, depression, and anxiety. Self-reported race and ethnicity were extracted from electronic health records. Weighted analyses were used to evaluate the association between racial and ethnic category and prenatal depression and anxiety; the prevalence of each stressor by race and ethnicity; and the relationship between each stressor and prenatal depression and anxiety in each racial and ethnic category.

**Results:**

The sample was 22% Asian, 3% Black, 20% Hispanic, 5% Other/Multiracial/Unknown, and 49% White. Compared to White people, Black and Hispanic people had a higher prevalence of prenatal depression (aPR: 1.85, 95% CI: 1.45, 2.35 and aPR: 1.17, 95% CI: 1.00, 1.37, respectively) and anxiety (aPR: 1.71, 95% CI: 1.34, 2.18 and aPR: 1.10, 95% CI: 0.94, 1.29, respectively). Compared to White people, Black and Hispanic people had a higher prevalence of moderate/severe distress due to changes in prenatal care (24 vs. 34 and 31%), and food insecurity (9 vs. 31 and 24%). Among Black and Hispanic people, distress due to changes in prenatal care was associated with a greater prevalence of prenatal depression (aPR: 2.27, 95% CI: 1.41, 3.64 and aPR: 2.76, 95% CI: 2.12, 3.58, respectively) and prenatal anxiety (aPR: 3.00, 95% CI: 1.85, 4.84 and aPR: 2.82, 95% CI: 2.15, 3.71, respectively). Additionally, among Hispanic people, high-risk employment and food insecurity were associated with a greater prevalence of prenatal depression and anxiety.

**Conclusions:**

This study identified racial and ethnic disparities in mental health for pregnant Black and Hispanic people. Distress due to prenatal care changes contributed to the observed disparities in prenatal depression and anxiety for Black and Hispanic people and food insecurity additionally contributed to the observed disparities for Hispanic people. Addressing distress due to changes to prenatal care and food insecurity specifically in Black and Hispanic people may help reduce the high burden of poor mental health and reduce observed disparities in these communities.

## Introduction

The COVID-19 pandemic in the U.S. has disproportionately impacted communities of color with substantially higher rates of infection, hospitalization, and death documented in Asian, Black, and Hispanic people compared to White people (1). In addition, the pandemic has exacerbated deep-seated social and economic disparities related to living conditions and work environments (2) putting communities of color at heightened risk of COVID-19-related stressors and adverse mental heath outcomes.

Pregnant people in particular, have been acutely impacted by the COVID-19 pandemic. Compared to White people, pregnant people of color are at greater risk of severe COVID-19 illness with Black and Hispanic people at highest risk (3). Changes to prenatal care, concerns about the heightened risks of SARS-CoV-2 infection for themselves and their fetus, and school closures and loss of childcare (4) have increased stress in pregnant people. Several COVID-19-related health and health care stressors (e.g., a job with increased risk of SARS-CoV-2 infection, distress over changes to prenatal care) as well as economic stressors (e.g., childcare challenges) have been reported to be associated with prenatal psychological distress (depression, anxiety) (5–9). Given the disproportionate impact of the pandemic on communities of color, pregnant Asian, Black, and Hispanic people may experience higher rates of COVID-19-related stressors as well as depression and anxiety, compared to pregnant White people.

While disparities in prenatal mental health have been documented prior to the pandemic with higher rates of depression and anxiety among pregnant Black and Hispanic people compared to White people (10, 11), little research on prenatal mental health disparities has been conducted during the pandemic (12). With the goal of identifying racial and ethnic disparities in mental health among pregnant people during the COVID-19 pandemic and COVID-19 pandemic-related contributors to these disparities, we followed the framework for disparity investigation described by Ward et al. (13) This framework shifts away from the sole use of interaction terms to assess health disparities and instead promotes a more comprehensive approach through a combination of racial and ethnic group-specific estimates in each of the following: (1) the prevalence of the outcome, (2) the prevalence of the exposure, and (3) the relationship between the exposure and outcome. Ward et al. demonstrate that an exposure associated with an outcome in the disadvantaged group can contribute to a disparity regardless of whether the relationship differs between racial and ethnic groups if differences in prevalence of the exposure are also taken into account. The contribution of this exposure to the disparity would be overlooked when relying only on significance testing of interaction terms without considering prevalence of the exposure. Thus, this framework allows for a more comprehensive and informative understanding of contributors to health disparities than that from use of interaction terms only. We hypothesized COVID-19-related stressors will contribute to racial and ethnic disparities in prenatal depression and anxiety.

## Methods

### Setting

This study was conducted in Kaiser Permanente Northern California (KPNC), a large integrated health care delivery system that provides comprehensive medical care to over 4.5 million members. All 15 regional service centers (with 48 associated office facilities) have Obstetrics and Gynecology and Behavioral Medicine/Psychiatry Departments. KPNC members include people covered by employee-sponsored insurance plans, the insurance exchange and Medicaid. Coverage is provided for ~40% of the northern California population. KPNC members are socio-demographically similar to the population living in the geographic area served by the health plan (14). KNPC maintains comprehensive electronic health records (EHR) for all members.

### Study Design

This cross-sectional analysis was conducted using data from the ongoing KPNC COVID-19 Pregnancy Study (15). This analysis includes data collected from pregnant KPNC members between June 22, 2020 and April 28, 2021. Detailed information on the study design and cohort has been published previously (15). Briefly, pregnant KPNC members at least 12 weeks of gestation, 18 years of age or older and English-speaking were identified from the EHR every 2 weeks and invited to complete a brief, web-based survey.

The Institutional Review Board of KPNC approved all study procedures and participants indicated informed consent by completing the survey after reviewing the consent information in the recruitment email.

### Measures

**Race and ethnicity** categories were ascertained from self-reported race and ethnicity in KPNC's electronic health records (EHR) and defined as Asian, Black, Hispanic, Other, and White. The Other category included racial and ethnic categories for which there were a small number of participants (Native American, Pacific Islander, Multiracial) as well as Unknown.

Prenatal mental health outcomes were captured in the survey. **Depression**. The 8-item, Patient Health Questionnaire (PHQ-8) depression screener has been validated for use in perinatal populations (16, 17) and was used to assess depression symptoms in the past 2 weeks. The PHQ-8 is similar to the PHQ-9 which has been validated for use across diverse racial and ethnic patients (18), with the exception that it excludes the question regarding suicide ideation. Scores range from 0 to 24 and scores of 10–24 were categorized as clinically significant depressive symptoms. **Anxiety**. The General Anxiety Disorder Scale (GAD-7) (19) has been validated in prenatal (20) and racially diverse populations (21, 22), and was used to measure anxiety symptoms in the past two weeks. Scores range from 0 to 21 and scores of 10–21 were categorized as clinically significant anxiety symptoms.

Several COVID-19 pandemic-related stressors were also captured in the survey. **Health and healthcare stressors**: Participants were asked if a healthcare provider had told them that they had or likely had COVID-19 (COVID-19 in pregnancy; y/n); if a member of their household had or probably had COVID-19 (Household member had COVID-19; y/n); whether their job put them at increased risk of COVID-19 (high-risk employment; y/n); and how distressed they were about changes to their prenatal care due to the COVID-19 pandemic (moderately/extremely vs. not at all/mildly). **Economic stressors**: All questions referred to the time frame of “since becoming aware of the COVID-19 pandemic.” Participants were asked if they lost their job permanently, temporarily or reduced their work hours (Lost job or reduced hours; y/n), if their spouse/partner lost their job permanently, temporarily or reduced their work hours (Partner lost job or reduced hours; y/n), and if their childcare was impacted such that they had difficulty arranging childcare or had to pay more for childcare (Childcare challenges; y/n). The validated 2-item Hunger Vital Sign screener was used to assess food insecurity (23). Food insecurity (y/n) was defined as responding often true or sometimes true (vs. never true) to either statement: (1) you worried that your food would run out before getting money to buy more or (2) the food you bought did not last and you did not have enough money to get more.

#### Demographic and Health Characteristics

The following data were ascertained from the EHR: maternal age at delivery (continuous in years), insurance type (commercial, government/public), trimester at survey completion (trimester one/trimester two, trimester three) and parity (zero, one, or 2+ previous births).

### Statistical Analysis

Inverse probability weights were created to account for survey non-response (20% response rate in the eligible sample; Supplementary Table 1). Inverse probability weights for survey response were calculated using predicted probabilities from a logistic regression model including variables expected to be associated with survey response (race and ethnicity category, insurance type, parity, trimester of pregnancy at the start of the COVID-19 pandemic), additional factors strongly associated with depression or anxiety symptoms (maternal age), and all interaction terms. Weights ranged from 1.56 to 59.19 in participants who completed the survey. To limit the influence from observations with extreme weights, weights were truncated at 20 for the 20 participants with weights >20 (24).

Multiple imputation by chained equations (MICE) (25) was used to address potential bias from excluding participants with missing data (complete case analysis) since data were not missing completely at random (26). We performed 100 imputations to impute missing values for people with missing data for covariates included in the regression models (14% missing parity, 1% missing insurance type). After imputation, we excluded participants with missing data for outcomes (*n* = 876) (27). The final analytic sample included participants with non-missing outcome information (*n* = 10,930).

The Ward et al. (13) framework for disparity investigation outlines three steps for identifying contributors to racial health disparities: (1) assess if there are differences in outcome prevalence (e.g., prenatal depression and anxiety) between racial and ethnic groups; (2) assess if there are differences in exposure prevalence (e.g., COVID-19-related health/healthcare and economic stressors) between racial and ethnic groups, and (3) evaluate whether the relationship between the exposure (COVID-19-related stressors) and outcome (prenatal depression and anxiety) differs between racial and ethnic groups. Conclusions about COVID-19-related stressors that contributed to racial and ethnic prenatal mental health disparities were based on the results from the analyses in accordance with the Ward et al. guidelines. Below we outline the methods used for each of these steps.

We first assessed racial and ethnic differences in the prevalence of prenatal depression and anxiety. According to the framework, establishment of a difference in the prevalence of the outcome is fundamental to the definition of a racial and ethnic health disparity. If there is no difference in the prevalence, then a disparity does not exist. We used weighted modified Poisson regression models (28) to estimate prevalence ratios and 95% confidence intervals for prenatal depression and anxiety associated with racial and ethnic group, with and without adjusting for potential confounders (maternal age, parity, insurance type, calendar month of survey completion, and trimester at survey completion) for each imputed dataset. Results were combined using Rubin's rules (29). The final point estimate was calculated as the average of the point estimate distribution across regression model results from each imputed dataset; the variance was calculated using both within- and between-imputation variances.

Second, we evaluated racial and ethnic differences in prevalence of COVID-19-related health/healthcare and economic stressors. Weighted percentages and chi square statistics were calculated for each COVID-19-related stressor by racial and ethnic group for each imputed dataset and combined across imputed datasets (30).

Third, we assessed whether the relationship between COVID-19-related stressors and prenatal depression and anxiety differed by racial and ethnic group. An association between the exposure and the outcome in the disadvantaged group was in some cases sufficient to conclude the stressor contributed to the disparity, depending on the prevalence of the exposure in the racial and ethnic groups (13). We used weighted modified Poisson regression models (28) to estimate prevalence ratios and 95% confidence intervals for prenatal depression and anxiety associated with each COVID-19-related stressor stratified by racial and ethnic category. Models were mutually adjusted for all COVID-19-related health/healthcare and economic stressors and potential confounders (maternal age, parity, insurance type, calendar month of survey completion, and trimester at survey completion). To test statistical differences in associations by racial and ethnic groups, we included a cross-product term between race and ethnicity category and each stressor in separate weighted modified Poisson regression models (28) for each stressor and prenatal depression and anxiety, adjusted for all COVID-19-related health/healthcare and economic stressors and potential confounders mentioned above. Tests for interaction generally have less power to assess statistical significance and thus a *p* < 0.10 is accepted as the cutoff for statistical significance for interaction terms (31). Models were run for each imputed dataset. Results were combined using Rubin's rules (29).

Analyses were conducted in SAS version 9.4 (SAS Institute INC., Cary, NC, USA) and R (version 4.1.2) (32).

## Results

The sample of 10,930 pregnant participants was 22% Asian, 3% Black, 20% Hispanic, 5% Other/Multiracial/Unknown, and 49% White (Table 1). Overall, a majority of the participants had private insurance (88%), completed the survey in the first or second trimester of pregnancy (79%), were an average 33 years of age, and had <2 previous births (67%). Black and Hispanic participants were more likely than Asian and White participants to have 2 or more previous births (24 and 23 vs. 11 and 12%, respectively) and have government/public insurance (23 and 13 vs. 5 and 7%, respectively).

**Table 1:** Demographic characteristics overall and by racial and ethnic category of 10,930 pregnant people seeking prenatal care at Kaiser Permanente Northern California during the COVID-19 pandemic between June 2020 and April 2021.

	**Overall (*n*, wtd %)**	**Asian**	**Black**	**Hispanic**	**Other/Unknown**	**White**	***p*-value**
**n**	**10,930**	**2,495**	**372**	**2,174**	**497**	**5,392**	
Age (mean, SE)	33 (0.04)	33 (0.1)	31 (0.3)	30 (0.1)	32 (0.2)	32 (0.1)	<0.001
Parity							<0.001
0	4,005 (31)	930 (32)	132 (28)	678 (26)	185 (30)	2,080 (34)	
1	3,755 (36)	903 (38)	98 (31)	694 (35)	130 (32)	1,930 (37)	
2+	1,626 (16)	267 (11)	84 (24)	510 (23)	50 (12)	715 (14)	
Missing	1,544 (17)	395 (18)	58 (17)	292 (16)	132 (26)	667 (15)	
Insurance type							<0.001
Government	559 (9)	75 (5)	60 (23)	207 (13)	29 (8)	188 (7)	
Commercial	10,214 (88)	2,394 (94)	306 (74)	1,930 (85)	445 (80)	5,139 (92)	
Missing	157 (2)	26 (1)	6 (3)	37 (2)	23 (12)	65 (2)	
Gestational age at survey							<0.001
1st/2nd trimester	8,328 (79)	1,916 (80)	264 (79)	1,603 (78)	386 (80)	4,149 (80)	
3rd trimester	2,602 (21)	579 (20)	98 (21)	571 (22)	111 (20)	1,243 (20)	

### Racial and Ethnic Differences in Prenatal Depression and Anxiety

#### Prenatal Depression

The overall prevalence of prenatal depression was 11%. There was considerable variation in prevalence of prenatal depression across the racial and ethnic groups, with the highest prevalence among Black and Hispanic participants (Table 2). Compared to White participants, Black participants had an 85% greater prevalence of prenatal depression [adjusted Prevalence Ratio (aPR): 1.85, 95% CI: 1.45, 2.35] after adjusting for potential confounders and Hispanic participants had a 17% greater prevalence of depression although only marginally statistically significant (aPR: 1.17, 95% CI: 1.00, 1.37). Additionally, prevalence of prenatal depression in Asian participants did not differ compared to White participants after adjusting for potential confounders (aPR: 0.94, 95% CI: 0.80, 1.11).

**Table 2:** Prevalence, crude prevalence ratios (cPRs) and adjusted prevalence ratios (aPR) evaluating the relationship between racial and ethnic category and prenatal depression and anxiety.

	**Prenatal depression**			**Prenatal anxiety**		
	**Prevalence *n* (wtd %)**	**cPR (95% CI)**	aPR^*^ (95% CI)	**Prevalence *n* (%)**	**cPR (95% CI)**	aPR^*^ (95% CI)
Asian (*n* = 2,495)	202 (8)	0.88 (0.75, 1.04)	0.94 (0.80, 1.11)	179 (7)	0.88 (0.75, 1.04)	0.79 (0.67, 0.94)
Black (*n* = 372)	72 (21)	**2.23 (1.76, 2.83)**	**1.85 (1.45, 2.35)**	66 (20)	**2.01 (1.57, 2.58)**	**1.71 (1.34, 2.18)**
Hispanic (*n* = 2,174)	263 (13)	**1.39 (1.20, 1.62)**	**1.17 (1.00, 1.37)**	265 (13)	**1.30 (1.12, 1.51)**	1.10 (0.94, 1.29)
Other/unknown (*n* = 497)	48 (10)	1.07 (0.79, 1.45)	1.02 (0.75, 1.38)	43 (9)	0.88 (0.63, 1.21)	0.84 (0.61, 1.17)
White (*n* = 5,392)	466 (10)	Ref	Ref	487 (10)	Ref	Ref

* *Adjusted for age (continuous), month of survey (continuous), trimester of survey, parity, insurance*.

*The bold values indicate statistically significant relationship*.

#### Prenatal Anxiety

The overall prevalence of prenatal anxiety was 11%. Black participants had a 71% greater prevalence of prenatal anxiety compared to White participants after adjusting for potential confounders (aPR: 1.71, 95% CI: 1.34, 2.18) (Table 2). Hispanic participants had a 10% greater prevalence of prenatal anxiety compared to White participants; however, the results did not reach statistical significance after adjusting for potential confounders (aPR: 1.10, 95% CI: 0.94, 1.29). In contrast, Asian participants had a lower prevalence of prenatal anxiety (aPR: 0.79, 95% CI: 0.67, 0.94) compared to White participants after adjusting for potential confounders.

### Racial and Ethnic Differences in COVID-19-Related Stressors

Overall, there were significant racial and ethnic differences in the prevalence of all health/healthcare and economic stressors (*p* < 0.001 for both). For the purpose of identifying COVID-19-related stressors that contribute to racial and ethnic disparities in prenatal mental health, we focus the text of this section on the racial and ethnic groups (Black and Hispanic) with a higher prevalence of prenatal depression or anxiety compared to White participants.

#### Health and Healthcare Stressors

**Figure 1:**
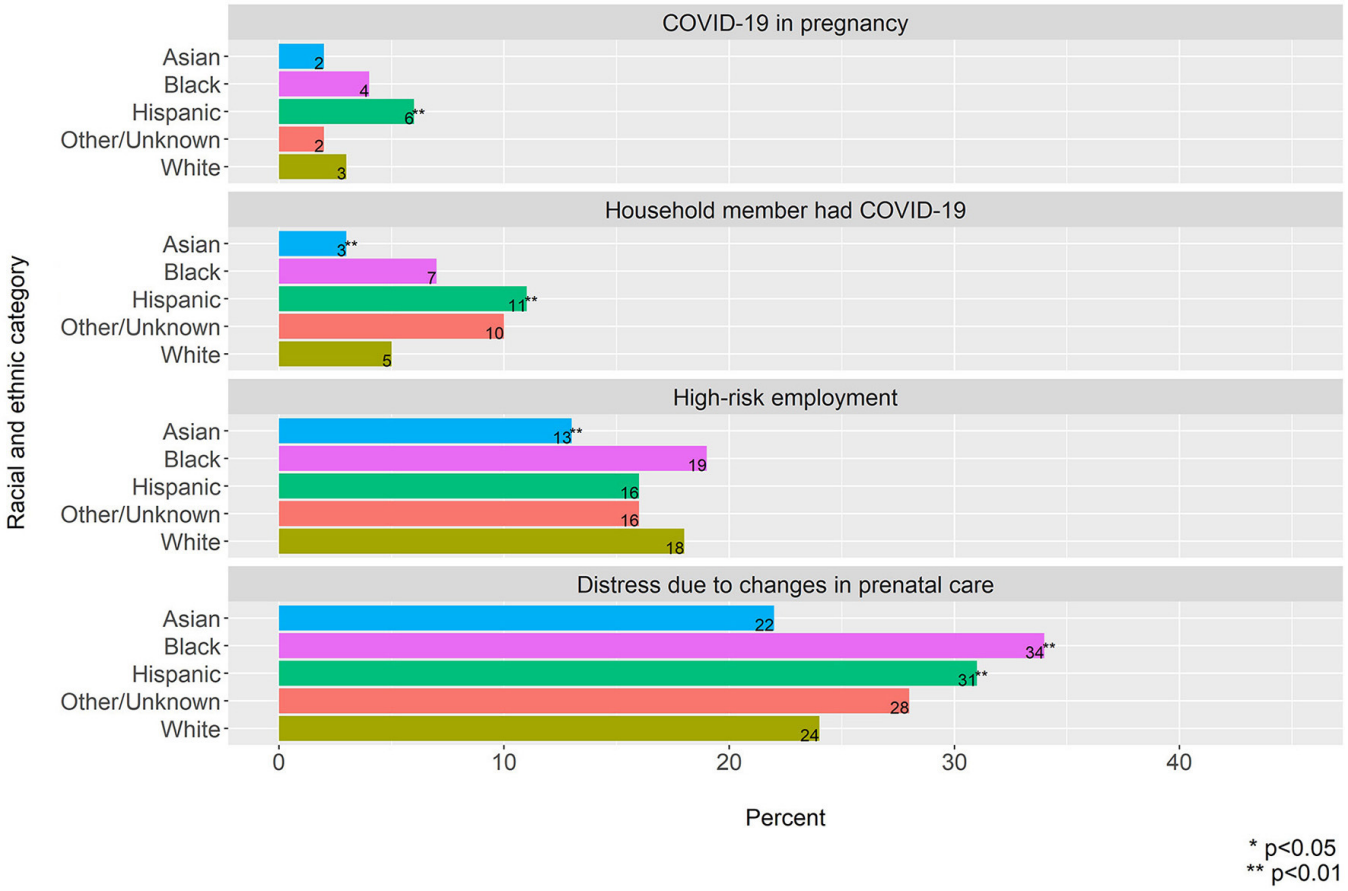
Prevalence of health and healthcare stressors by racial and ethnic category.

Black and Hispanic participants had a higher prevalence of distress due to changes in prenatal care compared to White participants (Black: 34 vs. 24%, *p* < 0.001 and Hispanic: 31 vs. 24%, *p* < 0.001) (Figure 1). Additionally, Hispanic participants had a higher prevalence of COVID-19 during pregnancy and a household member with COVID-19 compared to White participants (COVID-19 during pregnancy: 6 vs. 3%, *p* < 0.001, household member with COVID-19 11 vs. 5%, *p* < 0.001).

#### Economic Stressors

**Figure 2:**
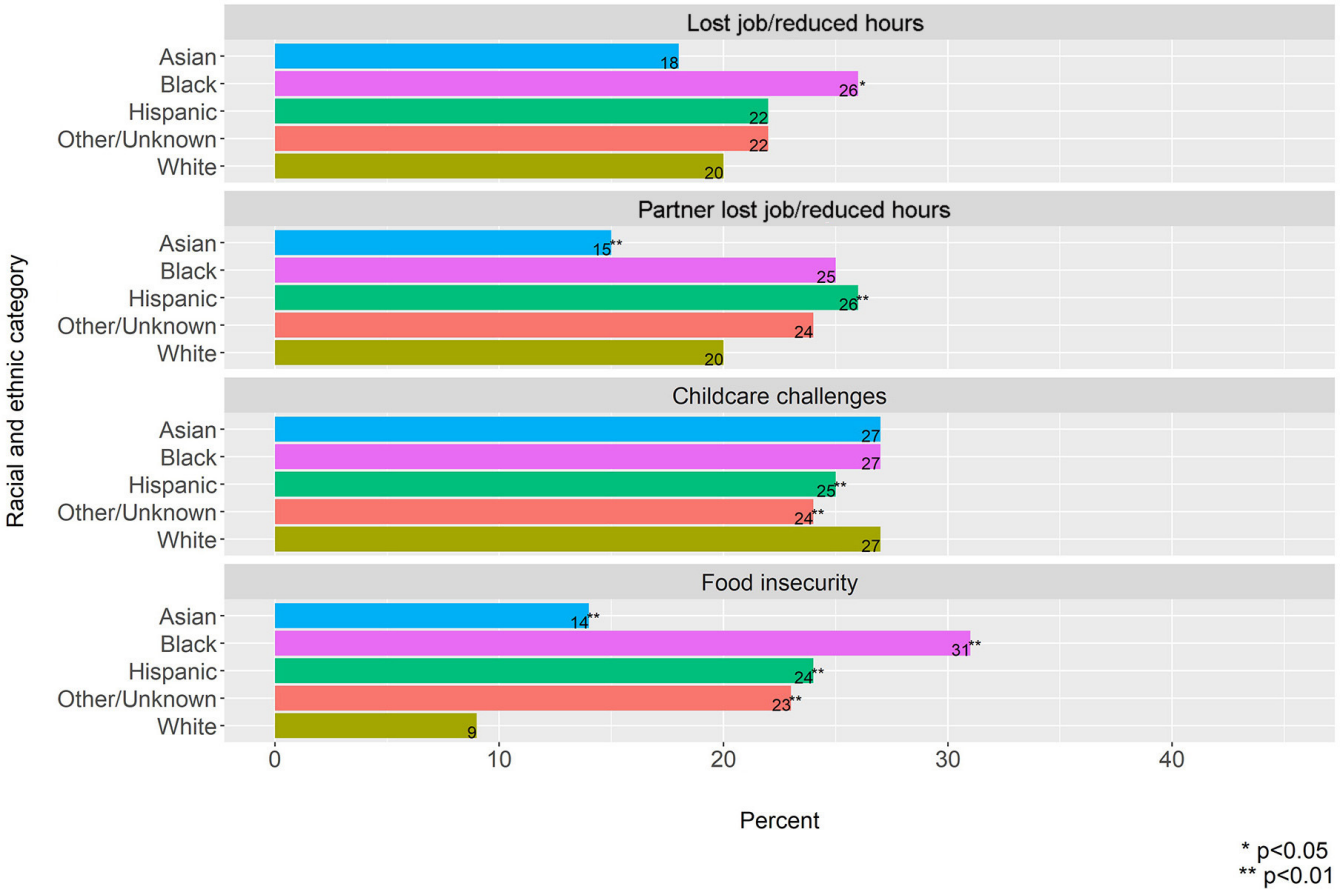
Prevalence of economic stressors by racial and ethnic category.

Black participants had a significantly higher prevalence of job loss compared to White participants (26 vs. 20%, *p* = 0.048) and although the prevalence for Hispanic participants was elevated compared to White participants it did not reach statistical significance (22 vs. 20%, *p* = 0.05) (Figure 2). On the other hand, Hispanic participants had a higher prevalence of having a partner lose their job compared to White participants (26 vs. 20%, *p* < 0.001) and while the prevalence was also elevated for Black participants it did not reach statistical significance when compared to White participants (25 vs. 20%, *p* = 0.08). Compared to White participants (9%), Black and Hispanic participants had a higher prevalence of food insecurity (Black: 31%, *p* < 0.001, and Hispanic: 24%, *p* < 0.001). There were no differences in childcare challenges across racial and ethnic groups.

### Associations of COVID-19-Related Stressors and Prenatal Depression and Anxiety by Racial and Ethnic Group

Similar to above, we focus the text of this section on racial and ethnic groups (Black and Hispanic) with a higher prevalence of prenatal depression and anxiety compared the White group.

Among Black participants, distress due to changes in prenatal care was the only stressor statistically significantly associated with prenatal depression (aPR: 2.27, 95% CI: 1.41, 3.64) (Table 3A). This association was similar to that in White participants (aPR: 2.41, 95% CI: 1.98, 2.92). Among Hispanic participants, high-risk employment (aPR: 1.32, 95% CI: 1.01, 1.71), distress due to changes in prenatal care (aPR: 2.76, 95% CI: 2.12, 3.58), and food insecurity (aPR: 1.70, 95% CI: 1.31, 2.19) were statistically significantly associated with prenatal depression. Associations of high-risk employment and distress due to prenatal care changes with prenatal depression were similar to those among White participants (high-risk employment aPR: 1.34, 95% CI: 1.09, 1.64; distress due to changes in prenatal care aPR: 2.41, 95% CI: 1.98, 2.92). The association of food insecurity with prenatal depression in White participants was stronger than that in Hispanic participants (aPR: 2.35, 95% CI: 1.83, 3.00).

**Table 3A:** Adjusted prevalence ratio (aPR) for the relationship between COVID-19-related health/healthcare and economic stressors and prenatal depression, stratified by racial and ethnic category.

	**Asian**	**Black**	**Hispanic**	**Other/unknown**	**White**
	***n* = 2,495**	***n* = 372**	***n* = 2,174**	***n* = 497**	***n* = 5,392**
	aPR^*^ (95% CI)	aPR^*^ (95% CI)	aPR^*^ (95% CI)	aPR^*^ (95% CI)	aPR^*^ (95% CI)
Health and healthcare stressors					
COVID-19 in pregnancy	2.11 (1.21, 3.69)	1.88 (0.74, 4.75)	1.35 (0.78, 2.31)	1.28 (0.24, 6.72)	1.12 (0.67, 1.87)
Household member had COVID-19	1.02 (0.58, 1.79)	1.02 (0.40, 2.60)	0.87 (0.55, 1.37)^**^	0.91 (0.28, 3.01)	**1.41 (0.99, 2.00)**
High-risk employment	1.41 (1.02, 1.94)	0.86 (0.50, 1.46)	1.32 (1.01, 1.71)	0.97 (0.48, 1.97)	1.34 (1.09, 1.64)
Distress due to prenatal care changes	2.51 (1.88, 3.34)	2.27 (1.41, 3.64)	2.76 (2.12, 3.58)	1.67 (0.85, 3.27)	2.41 (1.98, 2.92)
Economic stressors					
Lost job	0.98 (0.69, 1.40)	1.17 (0.72, 1.90)	1.00 (0.77, 1.30)^**^	3.35 (1.87, 5.99)^**^	**1.30 (1.05, 1.60)**
Partner lost job	1.29 (0.92, 1.81)	1.44 (0.90, 2.29)	1.15 (0.89, 1.48)	0.77 (0.38, 1.55)	0.91 (0.73, 1.14)
Childcare challenges	1.16 (0.80, 1.68)	0.97 (0.50, 1.86)^**^	1.24 (0.92, 1.67)	1.50 (0.71, 3.17)	**1.35 (1.06, 1.72)**
Food insecurity	2.30 (1.68, 3.15)	1.48 (0.92, 2.37)^**^	1.70 (1.31, 2.19)^**^	1.80 (0.90, 3.59)	**2.35 (1.83, 3.00)**

* *Additionally adjusted for age (continuous), month of survey (continuous), trimester of survey, parity, insurance*.

** *p < 0.10 for the cross-product term compared to the White racial category for the model that included all racial and ethnic groups*.

*The bold values indicate statistically significant relationship*.

Among Black participants, distress due to changes in prenatal care was the only stressor statistically significantly associated with prenatal anxiety (aPR: 3.00, 95% CI: 1.85, 4.84) (Table 3B). This association was similar to that in White participants (aPR: 2.89, 95% CI: 2.38, 3.50). Among Hispanic participants, high-risk employment (aPR: 1.56, 95% CI: 1.20, 2.02), distress due to changes in prenatal care (aPR: 2.82, 95% CI: 2.15, 3.71), and food insecurity (aPR: 1.66, 95% CI: 1.28, 2.15) were statistically significantly associated with prenatal anxiety. Associations of high-risk employment, distress due to prenatal care changes and food insecurity with prenatal anxiety were similar to those among White participants (high-risk employment aPR: 1.32, 95% CI: 1.08, 1.62; distress due to changes in prenatal care aPR: 2.89, 95% CI: 2.38, 3.50; food insecurity aPR: 1.84, 95% CI: 1.43, 2.36).

**Table 3B:** Adjusted prevalence ratio (aPR) for the relationship between COVID-19-related health/healthcare and economic stressors and prenatal anxiety, stratified by racial and ethnic category.

	**Asian**	**Black**	**Hispanic**	**Other/unknown**	**White**
	***n* = 2,495**	***n* = 372**	***n* = 2,174**	***n* = 497**	***n* = 5,392**
	aPR^*^ (95% CI)	aPR^*^ (95% CI)	aPR^*^ (95% CI)	aPR^*^ (95% CI)	aPR^*^ (95% CI)
Health and healthcare stressors					
COVID-19 in pregnancy	1.48 (0.71, 3.08)	1.57 (0.56, 4.39)	1.07 (0.61, 1.88)	2.23 (0.68, 7.31)^**^	**0.84 (0.45, 1.57)**
Household member had COVID-19	0.70 (0.32, 1.57)	1.39 (0.57, 3.36)	0.97 (0.62, 1.52)	1.97 (0.66, 5.88)	1.27 (0.86, 1.88)
High-risk employment	1.81 (1.30, 2.50)^**^	1.28 (0.79, 2.07)	1.56 (1.20, 2.02)	1.32 (0.67, 2.62)	**1.32 (1.08, 1.62)**
Distress due to prenatal care changes	3.69 (2.68, 5.09)	3.00 (1.85, 4.84)	2.82 (2.15, 3.71)	4.39 (2.28, 8.47)	2.89 (2.38, 3.50)
Economic stressors					
Lost job	0.86 (0.58, 1.27)	1.57 (0.99, 2.49)	1.05 (0.80, 1.37)	1.87 (0.90, 3.87)	1.10 (0.89, 1.37)
Partner lost job	1.04 (0.73, 1.51)	1.41 (0.90, 2.22)	1.12 (0.87, 1.44)	0.43 (0.17, 1.11)	0.97 (0.78, 1.21)
Childcare challenges	1.58 (1.03, 2.43)	1.18 (0.61, 2.27)	1.28 (0.94, 1.74)	1.64 (0.59, 4.57)	1.34 (1.05, 1.70)
Food insecurity	2.19 (1.59, 3.02)	1.47 (0.90, 2.38)	1.66 (1.28, 2.15)	1.50 (0.75, 3.01)	1.84 (1.43, 2.36)

* *Additionally adjusted for age (continuous), month of survey (continuous), trimester of survey, parity, insurance*.

** *p < 0.10 for the cross-product term compared to the White racial category for the model that included all racial and ethnic groups*.

*The bold values indicate statistically significant relationship*.

## Discussion

This study documented racial and ethnic disparities in prenatal depression and anxiety during the COVID-19 pandemic with a higher prevalence of both mental health outcomes in Black and Hispanic people compared to White people. Black and Hispanic people experienced a higher burden of COVID-19-related health/healthcare and economic stressors including distress due to changes in prenatal care, job loss, partner's job loss, and food insecurity compared to White people. Hispanic people additionally were disproportionately impacted by COVID-19 in pregnancy and having a household member with COVID-19, compared to White people.

While the increased risk of prenatal depression for pregnant Black people during the COVID-19 pandemic has been demonstrated previously (12), this study is the among the first to report an increased prevalence of prenatal anxiety among this group during the pandemic and an increased prevalence of prenatal depression and anxiety among Hispanic people. The higher rates of poor prenatal mental health among pregnant Black and Hispanic people compared to White people during the pandemic mirrors what has been documented prior to the COVID-19 pandemic (10). The consistently high burden of poor prenatal mental health in Black and Hispanic people documented in previous research and in our findings and the detrimental impacts of prenatal depression and anxiety on both mother and child, including greater risk of postpartum depression, (33, 34), preterm birth, small-for-gestational age (SGA) birth (35–37), and emotional and behavioral problems in the offspring (38, 39), highlight the importance of culturally appropriate, acceptable, and accessible interventions for prevention and treatment of prenatal depression and anxiety.

### Contribution of COVID-19-Related Stressors to Racial and Ethnic Disparities in Prenatal Depression and Anxiety

Applying the Ward et al. framework (13), one scenario where a stressor contributes to a disparity in a mental health outcome is when: (1) there is a disparity in the mental health outcome, (2) the stressor is more prevalent in the more disadvantaged racial and ethnic group compared to the White group, and (3) the stressor is associated with the mental health outcome in the more disadvantaged racial and ethnic group (regardless of whether that relationship is different from that in the White group). Thus, our findings suggest distress due to prenatal care changes contributed to the observed disparities in prenatal depression and anxiety for Black and Hispanic people and food insecurity additionally contributed to the observed disparities in prenatal depression and anxiety for Hispanic people. We note that if we had relied solely on the results from the interaction model, both distress due to changes to prenatal care and food insecurity would not have been identified as contributors to these disparities in prenatal mental health.

Although high-risk employment was also associated with greater prevalence of prenatal depression and anxiety in Hispanic people, the prevalence of high-risk employment and its association with prenatal depression and anxiety were similar in Hispanic and White people (Table 4). Thus, this indicates that high-risk employment is not a contributor to the observed disparities in prenatal depression and anxiety for Hispanic people. However, while it may not contribute to the noted disparity, employment with high-risk of exposure to COVID-19 has been associated with psychological distress in other studies in pregnant people (5) and other non-pregnant populations (7, 40). Fatigue, health worries, and fear have all been reported by people with high-risk employment even previous infectious disease outbreaks (41). Consideration of high-risk employment as a risk factor for psychological distress in pregnancy, especially during infectious disease outbreaks, should be considered in prenatal depression and anxiety screening strategies.

**Table 4:** Summary of approach for identifying contributors to observed racial and ethnic disparities in mental health outcomes in pregnant people during the COVID-19 pandemic.

**Mental health outcome**	**Observed disparity**	**COVID-19-related stressor associated with mental health outcome**	**Is there a difference in prevalence of the stressor by group?**	**Is there a difference in association of stressor and mental health outcome by group?**	**Stressor contributes to disparity**
Depression	Greater prevalence in Black people vs. White people	Distress due to prenatal care changes	Yes, higher prevalence in Black people	No, similar association in both groups	Yes
Depression	Greater prevalence in Hispanic people vs. White people	High risk employment	No, similar prevalence in both groups	No, similar association in both groups	No
		Distress due to prenatal care changes	Yes, higher prevalence in Hispanic people	No, similar association in both groups	Yes
		Food insecurity	Yes, higher prevalence in Hispanic people	Yes, stronger association in white people	Yes
Anxiety	Greater prevalence in Black people vs. White people	Distress due to prenatal care changes	Yes, higher prevalence in Black people	No, similar association in both groups	Yes
Anxiety	Greater prevalence in Hispanic people vs. White people	High risk employment	No, similar prevalence in both groups	No, similar association in both groups	No
		Distress due to prenatal care changes	Yes, higher prevalence in Hispanic people	No, similar association in both groups	Yes
		Food insecurity	Yes, higher prevalence in Hispanic people	No, similar association in both groups	Yes

The COVID-19 pandemic forced rapid implementation of hybrid models of care including both in-person and virtual prenatal care visits. Previous research has documented a significant relationship between COVID-19-related changes in prenatal care and greater psychological distress during pregnancy (6–9). In our study, Black and Hispanic people in particular experienced distress due to the changes in prenatal care which contributed to the higher burden of poor prenatal mental health in these groups. Previous research has found some people of color report negative interactions with the healthcare system during pregnancy, including experiences of racism, unmet information needs, and stressful interactions with all levels of staff (42–44). Thus, there is a need for better communication and careful listening during prenatal visits in pregnant people of color. During the COVID-19 pandemic, Black people reported worrying more than White people about having a good birthing experience and receiving good prenatal care (12), suggesting that COVID-19-related changes in prenatal care may have created challenges for patient-provider communication that further exacerbated existing disparities in quality of prenatal care between Black and White people. As healthcare systems continue to make changes to prenatal care and evaluate which care-delivery modifications to implement as standard prenatal care, providers and healthcare system leaders must remain focused on quality of patient-provider communication and must work with Black and Hispanic communities to identify ways to address stressful aspects of changes in prenatal care.

Similar to previous research during the pandemic in pregnant (12) and non-pregnant populations (45), Black and Hispanic people experienced the highest prevalence of food insecurity. Food insecurity is strongly associated with prenatal depression (46, 47) as well as long-term maternal and child health. (48) Our study demonstrated food insecurity contributed to disparities in prenatal depression and anxiety for Hispanic people. However, the association of food insecurity with prenatal depression did not reach statistical significance among Black people, which may be due to the small sample size. Regardless, these findings highlight the high burden of food insecurity and its contributions to poor maternal mental health during pregnancy in populations of color.

This is among the first studies that we are aware of that has evaluated the contribution of food insecurity to racial and ethnic disparities in prenatal mental health. Research prior to the COVID-19 pandemic has documented income and employment status as a contributor to these disparities (10). Yet in our study job loss or reduced work hours by the participant or their partner was not a contributor. It is possible that food insecurity is a greater contributor to these disparities and an important area for future research especially given the potential policy and clinical implications if so. Addressing food insecurity in pregnant people may have a significant impact on both their nutritional and mental health. The high prevalence of food insecurity for Black and Hispanic people during pregnancy highlights the importance of implementing universal screening for food insecurity in prenatal care and systems to connect people identified as food insecure with available federal and community resources.

### COVID-19-Related Stressors and Mental Health Outcomes in Pregnant Asian People

Although we did not identify a disparity in mental health outcomes for Asian people compared to White people, several COVID-19-related stressors, including high-risk employment, distress due to prenatal care changes, and food insecurity, were strongly related to prenatal depression and anxiety in Asian people. The prevalence of COVID-19-related stressors tended to be lowest among Asian people, which may explain why prevalence of depression and anxiety was also lowest in this group, even though there were strong associations of COVID-19-related stressors and mental health in Asian people. Future research should look at potential protective factors against poor prenatal mental health in Asian people, given the observed lower prevalence of depression and anxiety.

## Limitations and Strengths

Our study had some limitations that should be considered. Depression and anxiety outcomes were based on symptoms in the past two weeks at the time of survey completion and thus may not have captured people who completed the survey later in pregnancy but had high depression or anxiety symptoms earlier in pregnancy that resolved. Additionally, selection bias due to survey non-response is of concern given the low survey response rate. However, our analyses accounted for survey non-response by weighting analyses to account for differences between those who responded to the survey and non-responders. Current psychological distress may influence participation in the study; we were not able to directly compare mental health symptoms between those who participated in the study and those who did not. However, we found a similar prevalence of a history of an anxiety or mood disorder for people who did and did not complete the survey. We also accounted for potential bias due to missing data not at random by using multiple imputation. Although this study included more Black people than other studies assessing racial and ethnic differences in COVID-19-related stressors and prenatal mental health during the COVID-19 pandemic, the prevalence ratio estimates for associations of COVID-19-related stressors with prenatal depression and anxiety in Black people had wide confidence intervals, resulting in less precise estimates compared to the Hispanic and White people in this study. This limited the stressors we were able to investigate as potential contributors to observed prenatal mental health disparities in Black people. The relatively small sample of Black people who completed our study survey highlights the need for cohorts solely focused on Black individuals and oversampling of Black people in population-based studies to better understand contributors to prenatal mental health in this population. This also applies to the Native American, Pacific Islander, and Multiracial racial and ethnic categories that were combined due to small sample size within groups. We did not have information on cultural factors such as immigration status, social support or non-pandemic related socioeconomic factors beyond insurance type (such as education, income or marital status) that may be potential confounders. The study was limited to English-speaking people. The cross-sectional study design limits the ability to make causal conclusions and it is possible that prenatal psychological distress had an impact on COVID-19-related stressors. However, the mental health scales asked about symptoms in the past 2 weeks while the time frame for the COVID-19-related stressors reflected anytime since the start of the pandemic, diminishing the potential for reverse causation. While the study population from which this sample was drawn is socio-economically representative of the Northern California population living in the same geographic area served by Kaiser Permanente, generalizability to other populations may be limited.

Our study also has several strengths. This study addresses an important gap in the current literature, as research on racial and ethnic differences in COVID-19-related stressors and mental health in pregnant people has been limited. Additionally, a majority of previous research has been limited to data spanning the beginning of the COVID-19 pandemic, while this study covers almost 12 months of the COVID-19 pandemic. Finally, this study's representativeness of the entire KPNC population of pregnant people during the study period, and diversity across sociodemographic characteristics and stages in pregnancy at survey completion are further strengths of this study.

## Conclusion

This study highlights racial and ethnic disparities in mental health for pregnant Black and Hispanic people and identifies COVID-19-related health/healthcare and economic stressors contributing to these disparities. Continued screening for poor mental health coupled with appropriate interventions for all pregnant people during the COVID-19 pandemic is warranted, but addressing distress due to changes to prenatal care and food insecurity specifically in Black and Hispanic people may help reduce the high burden of poor mental health and reduce observed disparities in these communities.

## Data Availability Statement

The original contributions presented in the study are included in the article/Supplementary Material, further inquiries can be directed to the corresponding author.

## Ethics Statement

KPNC's Institutional Review Board approved all study procedures and women indicated informed consent prior to participation in the survey.

## Author Contributions

LA: concept and design and administrative, technical or material support. LA, NN, SB, KY-W, JA, YZ, MH, AF, OZ, and LC: acquisition, analysis and interpretation of data, and critical revision of manuscript for important intellectual content. LA, NN, and SB: drafting of manuscript. NN: statistical analysis. LC and AF: obtained funding. All authors contributed to the article and approved the submitted version.

## Funding

This work was supported by Community Health, Kaiser Permanente Northern California and the National Institutes of Health. LA was supported by the National Institute of Health (R01HD101483, R01DA047405, R01DA048033). JA and SB were funded by the Postdoctoral Training Program in Women's and Children's Health supported in part by Community Health, Kaiser Permanente Northern California. JA was additionally funded by the National Institute of Environmental Health Sciences (K99ES032481) and SB was additionally funded by the Eunice Kennedy Shriver National Institute of Child Health and Human Development (K99HD100585). YZ was supported by National Institute of Diabetes and Digestive and Kidney Diseases (K01DK120807). KY-W was supported by National Institute on Drug Abuse (K01DA043604, DA048033, and DA047405). OZ was funded in part by the National Institute of Allergy and Infectious Diseases (K01AI139275).

## Conflict of Interest

LA, NN, YZ, LC, KY-W, OZ, MH, AF, JA, and SB were employed by Kaiser Permanente Northern California.

## Publisher's Note

All claims expressed in this article are solely those of the authors and do not necessarily represent those of their affiliated organizations, or those of the publisher, the editors and the reviewers. Any product that may be evaluated in this article, or claim that may be made by its manufacturer, is not guaranteed or endorsed by the publisher.
