# Supplementary material for: Contributions of COVID-19 Pandemic-Related Stressors to Racial and Ethnic Disparities in Mental Health During Pregnancy
Source: Front Psychiatry. 2022 Mar 14;13:837659. doi: 10.3389/fpsyt.2022.837659 (PMC8964047; doi:10.3389/fpsyt.2022.837659)
Supplement: Supplementary file 1 [file Table_1.docx]

| **Supplemental Table 1. Characteristics of pregnant people with completed surveys compared to the overall invited population of pregnant people and those excluded for not completing the PHQ-8 or GAD-7** | | | |
| --- | --- | --- | --- |
|  | **Sample**  **n (%)** | **Non-responders**  **n (%)** | **Excluded due to missing PHQ-8 OR GAD-7**  **n(%)** |
| n | 10930 | 47199 | 876 |
| Age (mean, SE) | 33 (0.04) | 31 (0.02) | 33 (0.1) |
| Parity |  |  |  |
| 0 | 4005 (31) | 13735 (29) | 286 (33) |
| 1 | 3755 (36) | 15598 (33) | 320 (37) |
| 2+ | 1626 (16) | 9593 (20) | 158 (18) |
| Missing | 1544 (17) | 8273 (18) | 112 (1) |
| Insurance type |  |  |  |
| Government | 559 (9) | 4887 (10) | 53 (6) |
| Commercial | 10214 (88) | 40998 (87) | 808 (92) |
| Missing | 157 (2) | 1314 (3) | 15 (2) |
| Gestational age at survey |  |  |  |
| 1st/2nd trimester | 8328 (79) | 37699 (80) | 670 (76) |
| 3rd trimester | 2602 (21) | 8913 (19) | 206 (24) |
| Missing |  | 587 (1) | 0 (0) |
| Race |  |  |  |
| Asian | 2495 (25) | 11837 (25) | 232 (26) |
| Black | 372 (7) | 3851 (8) | 44 (5) |
| Hispanic | 2174 (27) | 13786 (29) | 190 (22) |
| Other/Multiracial/Unknown | 497 (6) | 3042 (7) | 48 (5) |
| White | 5392 (35) | 14712 (33) | 362 (41) |
| History of anxiety/mood disorders | 2691 (25) | 10392 (22) | 210 (24) |
